# The Pharmacopœia of the United States of America

**Published:** 1887-01

**Authors:** 


THE PHARMACOPŒIA OF THE UNITED STATES OF AMERICA.—Sixth Decennial Revision: By authority of the National Convention for Revising the Pharmacopœia, held at Washington, D. C., A. D. 1883.

To undertake a "Review" of The Pharmacopœia at this late day would be about like a criticism on Blackstone's Commentaries; and to give an opinion on its merits, would be about as presumptious. Nevertheless, we have thought it would not be amiss to point out, for the information of our readers, some of the important changes which have been made in this, the standard authority of strength and purity of drugs and medicines for the guidance of physicians who order, and druggists who dispense them; seeing that there are many old practitioners who are not aware that important ehanges have been made in medicines which they are daily prescribing. For instance, many suppose that Dover's Powder is still made with Sulphate Potassa; and some order the Chlorate or Bromide substituted; whereas there is no Potassa of any kind in the officinal powder,—Sugar of Milk having been substituted for the Sulphate Potassa,—both, however, are equally inert. Or, how many physicians are aware that Laudanum, as now made by the new U. S. P. is, by weight, ten, and by bulk, fifteen per cent stronger than formerly?

We don't know that we can accomplish our object better than by quoting the languageof *Lecture* 1, *of the National Institute oy Pharmach*, on the subject, as follows. Speaking of this revision, the *Lecture* says: There is

"1. An absolute alphabetical arrangement, dispensing with the so-called "secondary list."

2. The adoption of a nomenclature to conform to the more recent progress of botany, chemistry, etc., viz:

(*a*) The officinal Latin title of a vegetable drug to be the botanical genus name; i. e. *Erythroxylon*, instead of *Coca*.

(*b*) The officinal English title to be the scientific botanical rather than the vernacular,—i–e *Pilocarpus* instead of *Jaborandi*.

(*c*) The Latin names of alkaloids to terminate in *ina*, corresponding with *ine* in English, i–e–Morph*ina*—ine, Quin*ina*—ine, instead of Morphia, Quinia, etc. Neutral principles to terminate in *inum* English—*in*; i–e, Santon*inum*—in, etc.

2. The adoption of parts by weight, and the abandonment of all measures of capacity; qnantity being expressed by weight. This system unlike those formerly in use, specifies no definite quantity, but merely the quantity by proportion of different component parts. While this proportion is expressed in decimal parts, in order to harmonize with the metric (or decimal) system of weight, it nevertheless can be applied to any other system, such as avoirdupois,—provided that the same kind of weight is maintained throughout any operation involving its use."

A further resume of the changes which have been made since last revision (1870), shows that sixty-three, or about 20 per cent. of the primary list, and forty-five, or over half of the "secondary list," and one hundred and twenty "preparations" have been dismissed; and there remain now nine hundred and ninety-seven substances (crude drugs and preparations) which are enumerated and described. These were formerly divided into "Materia Medica" and "Preparations,"—the former class being either crude drugs, or were furnished by manufacturers and not prepared by the pharmacist: the latter class, composed of processes and formulas for making the "Preparations." In the revision this arbitray division has been dropped, and the *whole* are arranged alphabetically, as stated, which much facilitates references.

The required strength of opium, the most important drug perhaps in the list, has been largely increased ; it must now contain twelve to sixteen per cent. of morphia to be "officinal," against ten ("or over")per cent. in the older list.

Extract aconite is now made from the root instead of from the leaves, as formerly, and is therefore stronger ; while the tincture of aloes, is just three times the strength of former editions.

Quite a number of obsolete and unused drugs and preparations have been dismissed ; and a new department—Abstracta—has been added. These consist of dry powdered extracts, which are just twice the strength of the corresponding fluid extract.

There has also been added a "Table of Solubility," showing in what proportion certain drugs will dissolve in the various menstrua, a very important addition, and one which physicians would do well to study, since we are informed that many physicians prescribe amounts of salts and other drugs which will not dissolve in the amount of fluid ordered. This leads to embarrasment and delay. There has also been added to each important chemical described, a reliable test for purity.

Every live physician should have, and keep a copy of the U. S. Pharmacopœia for references. Do not get this work confounded with the vast, bulky dispensatory—it is a small, compact book of some 500 pages, and is gotten up in exquisite style by the house of Wm. Wood & Co. Price, $5.00.

